# Surgically Acquired Deficits and Diffusion Weighted MRI Changes after Glioma Resection - A Matched Case-Control Study with Blinded Neuroradiological Assessment

**DOI:** 10.1371/journal.pone.0101805

**Published:** 2014-07-03

**Authors:** Asgeir S. Jakola, Erik M. Berntsen, Pål Christensen, Sasha Gulati, Geirmund Unsgård, Kjell A. Kvistad, Ole Solheim

**Affiliations:** 1 Department of Neurosurgery, St. Olavs University Hospital, Trondheim, Norway; 2 MI Lab, Norwegian University of Science and Technology, Trondheim, Norway; 3 National Centre for Ultrasound and Image Guided Therapy, Trondheim, Norway; 4 Department of Radiology, St. Olavs University Hospital, Trondheim, Norway; 5 Department of Circulation and Medical Imaging, Norwegian University of Science and Technology, Trondheim, Norway; 6 Department of Neuroscience, Norwegian University of Science and Technology, Trondheim, Norway; The George Washington University, United States of America

## Abstract

**Background:**

Acquired deficits following glioma resection may not only occur due to accidental resection of normal brain tissue. The possible importance of ischemic injuries in causing neurological deficits after brain tumor surgery is not much studied. We aimed to study the volume and frequency of early postoperative circulatory changes (i.e. infarctions) detected by diffusion weighted resonance imaging (DWI) in patients with surgically acquired neurological deficits compared to controls.

**Methods:**

We designed a 1∶1 matched case-control study in patients with diffuse gliomas (WHO grade II–IV) operated with 3D ultrasound guided resection. 42 consecutive patients with acquired postoperative dysphasia and/or new motor deficits were compared to 42 matched controls without acquired deficits. Controls were matched with respect to histopathology, preoperative tumor volumes, and eloquence of location. Two independent radiologists blinded for clinical status assessed the postoperative DWI findings.

**Results:**

Postoperative peri-tumoral infarctions were more often seen in patients with acquired deficits (63% versus 41%, p = 0.046) and volumes of DWI abnormalities were larger in cases than in controls with median 1.08 cm^3^ (IQR 0–2.39) versus median 0 cm^3^ (IQR 0–1.67), p = 0.047. Inter-rater agreement was substantial (67/82, κ = 0.64, p<0.001) for diagnosing radiological significant DWI abnormalities.

**Conclusion:**

Peri-tumoral infarctions were more common and were larger in patients with acquired deficits after glioma surgery compared to glioma patients without deficits when assessed by early postoperative DWI. Infarctions may be a frequent and underestimated cause of acquired deficits after glioma resection. DWI changes may be an attractive endpoint in brain tumor surgery with both good inter-rater reliability among radiologists and clinical relevance.

## Introduction

Intracranial glioma surgery can be a delicate balance between risks and benefits. While extensive resections may prolong survival, [1], [2], [3] new deficits are associated with impaired quality of life [4] and possibly also shorter survival. [5], [6] While a range of imaging tools or stimulation techniques may facilitate the detection of tumor borders or functionally active tissue, [7], [8], [9], [10] acquired deficits may occur despite use of such advanced techniques because of peri-lesional infarctions. When increasing resection grades, the margins to functional brain will be shorter and as a consequence the potential for vascular iatrogenic damage can increase [11], [12].

Diffusion weighted magnetic resonance imaging (DWI) can detect infarctions early after onset, [13], [14], [15] but the clinical significance of infarctions as detected with DWI remains debated in glioma surgery. Ulmer et al. found abnormalities when using early postoperative DWI in 35 (70%) of 50 glioblastoma resections. In 6/10 patients with new neurologic deficits the deficits were possibly related to the area of diffusion abnormality. [16] In another study, rims of DWI abnormality around the cavity were seen after 31 of 80 (39%) tumor resections, but were considered to be normal postoperative changes. Non-rim DWI abnormalities were found in 15 (19%) of the 80 patients and in 9 (38%) of the 24 patients with surgically acquired deficits. In multivariate analyses non-rim DWI abnormalities were associated with impaired recovery in patients with deficits. [17] A very recent consecutive case series found DWI abnormalities to be present in 31% of all primary glioma resections, but in 50% among the patients with neurological deterioration (transient or permanent) [18].

Since DWI abnormalities after glioma surgery often are clinically silent the clinical relevance is not obvious. [16], [19] To our knowledge there are no balanced comparisons of MRI findings in patients with vs. without surgically induced deficits. Whether detection of DWI abnormalities is a reliable outcome measure and is associated with clinical outcome remains unknown. This needs to be answered before using DWI as a surrogate end-point for safety, as seen recently [20].

We designed a 1∶1 matched case-control study to examine the frequency, volume and characteristics of infarctions as detected by early postoperative DWI in glioma patients with and without acquired surgical deficits. Due to the variation in prevalence reported we also sought to assess the diagnostic reliability of early postoperative DWI findings after glioma surgery.

## Materials and Methods

### Study population

In this retrospective case-control study we screened all adult patients (≥18 years) operated for diffuse gliomas with surgical resection at our institution from January 2004 through February 2012. The patients were operated in general anesthesia with a strategy combining import of MRI data into a navigation system with intraoperative 3D ultrasound. [21], [22] Resections were performed according to these tumor margins in the 3D ultrasound images, with guidance of fMRI and DTI in eloquent areas, as reported earlier. [21] To correct for the brain shift caused by the resection, the 3D ultrasound acquisition was repeated several times during the operation.

Surgical morbidity was assessed retrospectively from medical records. Patients who underwent biopsy only were excluded. Consecutive patients with new or worsened early postoperative language and/or motor deficits of any magnitude were included as cases. We decided to use these two types of deficits as these presumably are robust for retrospective assessment. Patients with acquired deficits that could be related to surgical complications (e.g. postoperative hematoma, subdural empyema) were excluded. A matching procedure to identify controls was undertaken, blinded for postoperative MRI findings. Control patients were without registered acquired deficits and matched 1∶1 with cases with respect to preoperative tumor volumes (in quartiles of the entire glioma population), histopathological grade (i.e., WHO high- or low-grade glioma), and anatomical eloquence as defined by Sawaya. [23] Tumor volumes and resection grades were determined from preoperative and early postoperative MRI volumes using an ellipsoid model (4л×r^3^/3), as described by others. [24] Gross total resection (GTR) was defined as no visible contrast enhancing tumor tissue on the early (<72 hours) postoperative 1.5 T or 3.0 T MRI scans in contrast enhancing tumors. In tumors not demonstrating contrast enhancement GTR was defined as removal of all hyperintense changes on T2 or FLAIR images.

### Diffusion weighted imaging

**Figure 1:**
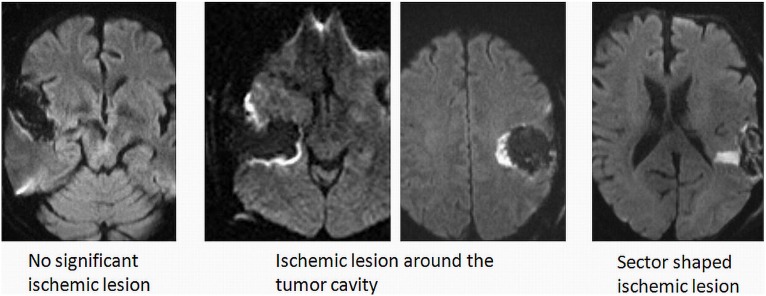
Example of the different main categories of ischemic lesions used in this study.

The DWI technique relies on the motion of water molecules reflecting the micro-environmental architecture. [25] In acute brain infarction water molecule diffusion is restricted due swelling of the cells, i.e. cytotoxic edema. Areas of reduced diffusion will appear with high signal on DWI images and low signal on the corresponding apparent diffusion coefficient (ADC) maps. Our protocol is that all patients undergo routine preoperative and early postoperative (<72 h) MRI examinations performed on either 1, 5T or 3T MRI systems. DWI is included in postoperative MR examinations as a part of the diagnostic routine. A single shot, spin echo echo-planar imaging sequence was used. Diffusion gradients were applied in three orthogonal directions to generate isotropic DWI. On 1, 5T (Siemens Avanto) the sequence parameters were: TR/TE = 3000/89 msec., field of view (FOV) 23 cm, matrix 192×200, b values of 0, 500 and 1000 s/mm^2^ and slice thickness/gap = 5/1, 5 mm. On 3T (Philips Intera 3T) the sequence parameters were: TR/TE = 4542/60 msec., FOV 23 cm, matrix 124×97, b values of 0 and 1000 s/mm^2^ and slice thickness/gap = 3/0, 3 mm. On 3T (Siemens Trio) the sequence parameters were TR/TE = 2900/91 msec, FOV 23 cm, matrix 192×192, b values of 0, 500 and 1000 s/mm^2^ and slice thickness/gap = 5/1, 5 mm. In all examinations ADC maps were automatically calculated. In the present study we used relative apparent diffusion coefficient (rADC) values of 0.7 as cut-off for ischemia with higher values per definition not considered related to ischemia. The rADC was calculated as the ratio between ADC in ischemic and healthy brain tissue (rADC = ADC ischemia/ADC healthy). The cut-off was chosen based on previous studies on time course of ADC in brain ischemia. [26] The diffusion images were examined to detect post-operative ischemic changes by identifying areas with restricted diffusion (i.e. high signal on b = 1000 images and low signal on ADC-maps). To exclude diffusion abnormalities related to methemoglobin, areas of DWI hyperintensity that could not clearly be distinguished from T1-weighted hyperintense blood products were scored as no DWI abnormality. Available anatomical sequences and preoperative examinations were used to exclude other possible sources to the diffusion changes (e.g. abscess, artifacts or residual tumor). Areas with restricted diffusion were segmented manually on each slice by two radiologists familiar with the procedure. The volumes were calculated by adding the areas with diffusion restriction from each slice and multiplying with the slice thickness (including the interslice gap). In addition, the types of DWI change were registered as rim (surrounding cavity, small arterioles and excessive pressure), sector (arterial damage) and combined (combination of previous, but also including venous congestion), see figure 1. All inter-rater analyses on DWI data are from this first assessment. In cases with disagreement whether significant changes was present and cases with difference in volume larger than 30% of mean volume were re-assessed by an experienced neuroradiologist (K.A.K.). In such cases, the volume, type of changes and rADC values were dictated by this final measurement. DWI volumes used in analyses of neurological outcome were either mean volume when agreement was present or the volume from the review when discordant results were present in the first assessment. All radiologists were blinded for clinical characteristics, and had no information on whether patients were cases or controls.

### Statistics

All analyses were done with the PASW statistics, version 18.0. Statistical significance level was set to P<0.05. Q–Q plots were used to test for normal distribution of data. Central tendencies are presented as means if data is normally distributed and as medians when skewed. Kappa-statistics were utilized for categorical inter-rater analyses to determine the reliability of postoperative DWI changes after glioma resection (significant infarction present (yes/no) and types of infarction (rim/sector/combined)). The difference in DWI volumes (when agreement of diffusion changes was present) was tested with one sample t-test. Bland-Altman plot was used to assess bias in volume measurements in cases where it was agreement that significant changes were present. [27] We also explored which type of circulatory change that was most often seen in patients with acquired deficits compared to controls.

To achieve 80% power we estimated that 45 patients in each group were necessary to detect a difference in mean DWI abnormality volumes from 4.0 ml in cases to 2.5 ml in controls using standard deviation of 2.5 ml in each group. We believed that detecting a smaller difference than 1.0 ml would probably not be clinical useful due to such small volumes are difficult to separate in regular clinical use. The presented 42 patients in each group constitute a power of 78% for detecting the aforementioned difference.

### Ethics and approvals

The study protocol was approved by the Regional Ethical Committee for Health Region Mid-Norway and the need for informed consent was waived by the committee.

## Results

We identified 42 patients eligible for inclusion as cases and they were matched 1∶1 with 42 controls. The cases and controls are presented with the matching criteria in table 1 together with other baseline characteristics. However, after inclusion and radiological assessment it was noted that 2 cases were missing DWI sequences and these were consequently excluded from further analyses. Among the 40 cases with deficits 15 (38%) had mild deficits and 8 (20%) had temporary deficits.

**Table 1:** Baseline and treatment characteristics.

Baseline	Cases (N = 42)	Controls (N = 42)	p-value
Age, mean ± SD	54.5±14.1	56.2±12.4	0.540
Female (%)	14 (33%)	20 (48%)	0.182
Histopathology*			0.786
Diffuse grade II gliomas	8 (19%)	9 (21%)	
Diffuse grade III and IV gliomas	34 (81%)	33 (79%)	
Primary operation (%)	23 (55%)	32 (76%)	0.167
Preoperative contrast enhancement (%)	36 (86%)	34 (81%)	0.558
Tumor volume (cm^3^) mean ± SD	32.1±34.7	29.4±24.8	0.680
Tumor volume in quartiles*			0.491
1: range (0.73–32.85 cm^3^)	30 (71%)	29 (69%)	
2: range (36.99–63.20 cm^3^)	6 (14%)	8 (19%)	
3: range (69.55–95.45 cm^3^)	4 (10%)	5 (12%)	
4: range (128.34–162.87 cm^3^)	2 (5%)	0	
Sawaya grade of eloquence (%)*			0.999
Not eloquent	8 (19%)	8 (19%)	
Intermediate	15 (36%)	15 (36%)	
Eloquent	19 (45%)	19 (45%)	
Left sided tumor (%)	24 (60%)	23 (55%)	0.668
Preoperative symptoms†			
Seizures (%)	15 (36%)	14 (33%)	0.818
Motor deficit(s) (%)	18 (43%)	10 (24%)	0.143
Nausea & vomiting (%)	3 (7%)	4 (10%)	0.693
Visual (%)	3 (7%)	9 (21%)	0.061
Dysphasia (%)	7 (17%)	8 (19%)	0.776
Cognitive (%)	12 (29%)	8 (19%)	0.306
Dizziness & Ataxia (%)	9 (21%)	9 (21%)	1.000
Resection grade in %, median (IQR)	96.4 (84.5–100)	98.5 (90.4–100)	0.202
Gross total resection (%)	13 (31%)	19 (45%)	0.178

*Matching criteria.

† More than one symptom may be registered per patient.

### DWI volumes and clinical outcome

As seen in table 2, DWI changes were more often seen in patients with acquired deficits (63% versus 41%, p = 0.046), and median volumes of DWI abnormalities were larger in cases than in controls (median 1.08 cm^3^ versus 0 cm^3^, p = 0.047).

**Table 2:** DWI changes in cases and controls.

	Cases (N = 40)	Controls (N = 42)	p-value
Significant DWI changes present (%)	25 (63%)	17 (40%)	0.046
Volume DWI changes (cm^3^), median (IQR)	1.08 (0–2.39)	0 (0–1.67)	0.047†
Type of DWI changes			0.177
Rim (%)	14 (35%)	11 (26%)	
Sector (%)	6 (15%)	2 (5%)	
Combined (%)	5 (12%)	4 (9%)	
None (%)	25 (38%)	15 (60%)	
Volume rim DWI changes (cm^3^), median (IQR) (n = 25)	1.94 (1.28–2.90)	1.67 (0.69–2.28)	0.511
Volume sector/combined DWI changes (cm^3^), median (IQR) (n = 17)	2.15 (1.10–17.97)	4.78 (1.80–6.55)	0.841
Volume DWI changes >4 cm^3^	7 (18%)	5 (12%)	0.474
rADC‡ value,* mean ± SD	0.5±1.1	0.5±1.4	0.959

*When DWI changes were present.

† Analyzed with respect to overall difference in volume, not for subgroups were descriptive only in those with significant DWI changes are presented.

‡ rADC denotes relative apparent diffusion coefficient.

DWI abnormalities with only a rim pattern was seen in 35% of cases versus 25% of controls while DWI abnormalities other than only a rim around the cavity was observed in 27% of cases compared to 14% of controls (table 2). However, these difference did not reach statistically significance (p = 0.177).

Because specifically asked for in the review process we performed a post-hoc assessment of a potential link between deficits and DWI abnormalities in individual patients and classified the link as “possible” or “unlikely”. Of the 25 cases with DWI abnormalities 21 (84%) had DWI abnormalities that could possibly explain the deficit while 4 (16%) cases had an unlikely association between image findings and deficits.

### Interobserver variability of DWI analysis

As seen in table 3 there was complete agreement between radiologist concerning the presence or absence of DWI abnormalities in 67/82 patients (82%, substantial agreement with κ = 0.64, p<0.001). A second MRI review was needed in 29 patients (35%) due to differences in interpretation (absolute difference in DWI volume divided by mean volume >30% or disagreement if DWI changes were absent or present).

**Table 3:** Kappa statistics on presence or absence of DWI changes postoperatively (cases n = 40, controls n = 42).

Reviewer#2Reviewer#1	Not significant DWI changes	Significant DWI changes	Total
Not significant DWI changes	35	4	39
Significant DWI changes	11	32	43
Total	46	36	82

**Figure 2:**
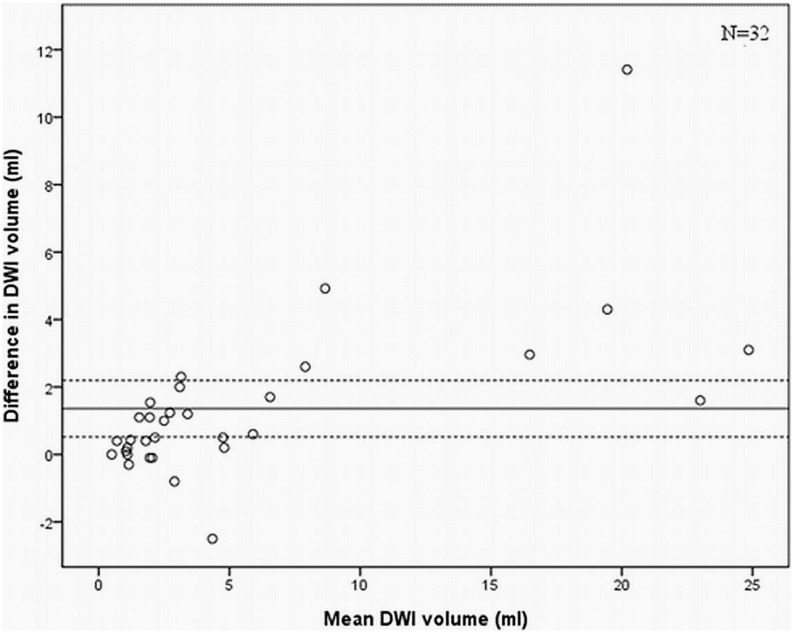
Bland Altman plot on DWI volume between observers demonstrating good overall agreement, but a slight tendency towards larger differences in DWI in larger lesions with vertical spread of scatter points being narrower at low values of mean than at high values of mean. This variation indicates that the method of manual segmentation of postoperative DWI changes depends somewhat on the volume of DWI changes. The line represents mean difference and the dotted lines the 95% CI.

There was a significant difference in DWI volume as measured by the two radiologists in the cases where there was an agreement that DWI abnormalities were present (N = 32, p = 0.003). The mean difference in volume measurements was 1.4 cm^3^ (95% CI 0.5–2.2) and in figure 2 the interobserver variability in volume measurements is illustrated with a Bland-Altman plot.

## Discussion

In this case-control study with blinded radiological assessment we demonstrate that there is a significant association between new neurologic deficits and infarctions as detected by early postoperative DWI following glioma surgery. Avoidance of microvascular injuries may be a key to reduce the chance of acquired deficits after surgery. Since 50% more ischemic lesions were seen in cases compared to matched controls this indicates that infarctions around resection cavities are a common and probably underestimated cause of surgically induced deficits. When aiming for radical resections the importance of such microvascular injuries may increase.

DWI changes after brain tumor surgery have so far not been extensively studied, and to our knowledge there has been no attempt to assess DWI changes in comparable groups. Reported frequencies of DWI alterations and alleged clinical significance vary between publications and imbalances of possible confounders such as lesion size, lesion type, and location make interpretation of case series difficult. Earlier publications also do not explore the reliability of diagnosing the reported postoperative DWI abnormalities, an important step in the evaluation of any outcome measure. In a mixed brain tumor population with both intra- and extraaxial lesions impaired recovery was reported if acquired deficit “could be explained by DWI changes”, but DWI alterations were also present in 11% of patients without acquired deficits. [17] However, due to the functional diversity of the brain, postoperative speculations about the functional importance of DWI lesions in various locations can be difficult in many cases. Another study found early postoperative DWI abnormalities in 64% of patients with diffuse gliomas (WHO grade II–IV), although detected lesions were considered to be clinically silent. [19] A recent study that excluded the small ischemic rims around the resection wall cavity found ischemic lesions in 31% after primary glioma resections while 50% of the patients with acquired deficits had ischemic lesions. [18] It was also observed that 80% of patients with recurrent gliomas had ischemic lesions, and that new deficits were more common after recurrent glioma operations. [18] However, another study reported no difference in the frequency of DWI abnormalities after primary as compared to recurrent glioma operations. [20] Volumes of DWI abnormalities have not been much studied, but in a study in low grade gliomas, the median volumes of infarctions as detected by DWI were 6.1 cm^3^ and it was reported that DWI abnormalities could lead to an overestimation of residual tumor volumes, especially in FLAIR sequences. [28] Our balanced and blinded case-control study significantly strengthens the evidence of the link between DWI abnormalities and deficits at a group level and thus also strengthens the clinical significance of such image findings. Peritumoral infarctions are clearly a common cause of postoperative neurologic deficits. We also found substantial agreement between radiologists in diagnosing postoperative DWI alterations, making DWI changes an attractive endpoint in brain tumor surgery with both good inter-rater reliability among radiologists and clinical relevance.

An open question is still whether iatrogenic peri-lesional strokes may increase the risk of postoperative seizures. If extrapolating data from stroke patients, infarctions will lead to early onset seizures in only 3% of patients. [29] Also, the reviews examining anticonvulsants administered prophylactically have failed to demonstrate any clear benefit in seizure prevention. [30], [31], [32] Thus, in brain tumor surgery the evidence is too weak for recommending prophylactic anticonvulsant treatment solely because of postoperative infarctions as detected by DWI.

Although we observed DWI changes in 40% of controls (65% of these were rim changes only), it should be remembered that nearly 60% of patients were without such ischemic changes. Thus, surgery without ischemic lesions as detected by DWI is clearly possible and should be a research focus. How are tumors to be removed to avoid peri-tumoral infarctions? While there are several imaging tools and neurophysiological mapping techniques that may be helpful in detecting tumor borders and identifying functional brain tissue to reduce the chance of removal of functional brain tissue during surgery, these are not necessarily helpful for avoiding microvascular injuries. We use 3D ultrasound guided surgery, and 3D ultrasound angiography for intraoperative detection of vessels. This application is sensitive for identifying most vessels. However, it still needs to be improved to be able to detect small, deep-seated vessels like the lenticulostriate arteries. Another potential technique is perfusion ultrasound with the use of microbubbles, however to our knowledge this is not a technique that is refined for quantifying brain perfusion. [33] Whether ultrasound perfusion modalities in the future can be used for avoidance of peri-lesional infarctions or merely to visualize tumors, tumor vascularity or to detect infarctions after they have occurred remains to be seen. An indirect approach using continuous motor evoked potential registration may be of benefit in lesions affecting the internal capsule. [34] Changes in potentials can make the neurosurgeons take action and by movement of spatulas, irrigation or addition of spasmolytic agent, enable potential recovery. However, even awake surgeries with cortical and subcortical mapping may result in neurological deterioration in approximately 20% with eloquent tumors, with sustained long-term disability in 7%. [35] When operating a glioma near eloquent brain even a very small infarction extending only a few millimeters may cause a deficit. [36] This is perhaps one reason why the “1 cm rule”, leaving a margin around functional sites detected with mapping, has been widely used. [12] A key to further improve surgical results may be systematic studies on the causes of DWI abnormalities and factors that lessen the risk of infarctions. Not to be forgotten is that the surgical technique in terms of tissue handling, dissection technique, surgical approaches and modality of tumor removal (e.g. suction or bipolar coagulation versus ultrasonic aspirator) together with haemostasis strategies (e.g. irrigation, bipolar coagulation, and use of haemostatic agents) may play an important role when it comes to risk of acquired deficits due to microvascular injuries. Also, positioning to avoid unwarranted traction or need for spatulas may perhaps be important.

In stroke management DWI has proved useful at early time-points due to a high sensitivity and specificity. [13], [14], [37], [38] During the very first hours of cerebral ischemia the accuracy of DWI and the inter- and intraobserver reliability is excellent. [15], [39] We demonstrate a substantial correlation among radiologists when evaluating the presence or absence of significant infarction. Small differences in volume measurements were seen, but agreement was still generally good. The difference in test properties compared to assessment in patients with ischemic stroke is not unexpected since the assessment of postoperative DWI changes in tumor patients is naturally more complex. [28] Since postoperative infarctions that occur in critical regions predict long-term loss of function [17] it may be a useful objective surrogate quality marker for safe surgery (marker for neurological preservation), similar to resection grades for effective surgery (marker for improved survival or progression free survival). The implementation of routine DWI should not represent a significant challenge to neurosurgical departments already doing early postoperative MRIs. Early postoperative DWI is also important for the interpretation of subsequent images in intra-axial lesions as post-infarct enhancement may be misinterpreted as progressive tumor or malignant transformation. [16], [19].

It may be argued that the frequent finding of infarctions with DWI without detected neurological deficits is a limitation for use as a quality measure in single patients. Still to be acknowledged, standard clinical assessments of deficits from brain surgery are presumably insensitive. Although DWI changes in non-eloquent locations may not cause motor or language deficits, there are presumably no silent brain regions.

The matched case-control design and blinded assessment of postoperative MRI represent the main strengths of the present study. This study also has limitations. Most importantly the presence of new deficits was determined retrospectively. Even though we took precautions to avoid external factors (e.g. infection or hemorrhage) explaining neurological findings postoperatively it remains a possibility that some patients, at least those with transient deficits, could be explained by other undetected causes (e.g. seizures). Also, we can not exclude the possibility that some patients included as controls may have had subtle, but undetected, deficits. Limited by the number of patients with surgically induced deficits, statistical power was also marginal, perhaps explaining why differences between different types of DWI abnormalities were statistically insignificant in the subgroup analyses. In addition, our procedure to exclude diffusion abnormalities related to methemoglobin could theoretically lead to misinterpretation of infarctions with hemorrhagic transformation. However, reperfusion is a key step in hemorrhagic transformation and this is possibly less likely to occur in as a result of surgery. Also, hemorrhagic transformation usually occur in larger infarctions, in infarctions with cardioembolic etiology and with the use of drugs to inhibit coagulation, [40], [41] all being rare in this patient population end therefore this potential limitation should be very small.

## Conclusions

Postoperative infarctions as detected by DWI are more common in patients with new neurologic deficits after glioma surgery than in matched controls without acquired deficits. Patients with surgically acquired deficits also have larger postoperative infarctions compared to controls. The interoberserver variability among radiologists was acceptable for detecting DWI abnormalities and quantifying the size of infarction. Early postoperative DWI findings may have potential as a surrogate objective quality marker for safe and gentle brain surgery.
